# Double rolling circle replication (DRCR) is recombinogenic

**DOI:** 10.1111/j.1365-2443.2011.01507.x

**Published:** 2011-05

**Authors:** Haruko Okamoto, Taka-aki Watanabe, Takashi Horiuchi

**Affiliations:** 1 Department of Basic Biology, School of Life Science, The Graduate University for Advanced Studies (SOKENDAI)Myodaiji, Okazaki 444-8585, Japan; 2 Division of Genome Dynamics, Department of Basic Biology, National Institute for Basic Biology, School of Life ScienceMyodaiji, Okazaki 444-8585, Japan; 3 Department of Biosystems Science, School of Advanced SciencesSOKENDAI, Shonan Village, Hayama, Kanagawa 240-0193, Japan

## Abstract

Homologous recombination plays a critical role in maintaining genetic diversity as well as genome stability. Interesting examples implying hyper-recombination are found in nature. In chloroplast DNA (cpDNA) and the herpes simplex virus 1 (HSV-1) genome, DNA sequences flanked by inverted repeats undergo inversion very frequently, suggesting hyper-recombinational events. However, mechanisms responsible for these events remain unknown. We previously observed very frequent inversion in a designed amplification system based on double rolling circle replication (DRCR). Here, utilizing the yeast 2-μm plasmid and an amplification system, we show that DRCR is closely related to hyper-recombinational events. Inverted repeats or direct repeats inserted into these systems frequently caused inversion or deletion/duplication, respectively, in a DRCR-dependent manner. Based on these observations, we suggest that DRCR might be also involved in naturally occurring chromosome rearrangement associated with gene amplification and the replication of cpDNA and HSV genomes. We propose a model in which DRCR markedly stimulates homologous recombination.

## Introduction

**Figure 1:**
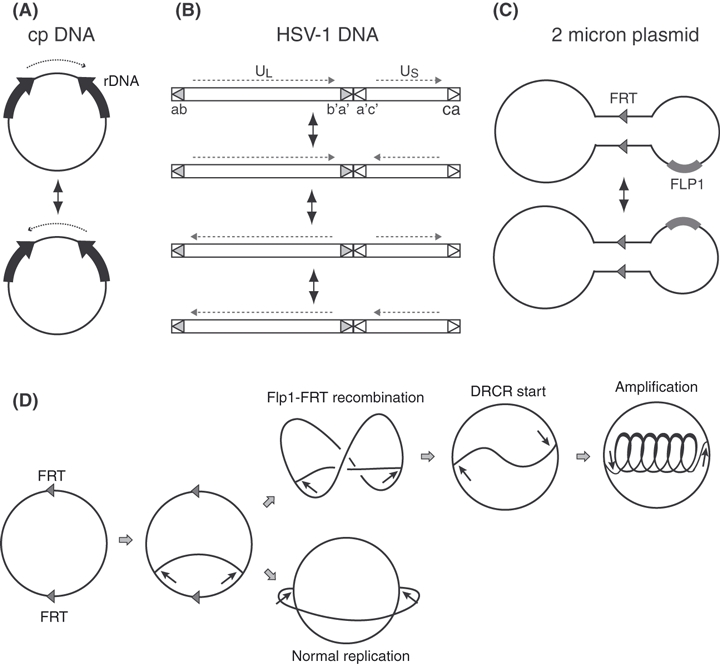
Isomeric structures of chloroplast (cp) DNA, HSV-1 DNA, 2-μm plasmid DNA and two modes (normal and DRCR) of replication in 2-μm plasmids. (A) Two isoform structures of chloroplast (cp)DNA, (B) four isoform structures of HSV-1 DNA, (C), two isoform structures of yeast 2-μm plasmid, (D) two modes of replication of 2-μm plasmids, normal and double rolling circle replication (DRCR).

Homologous recombination plays a central role in processes involved in genome instability, such as chromosomal rearrangements, gene diversification and molecular evolution, as well as in genome stability. In nature, interesting examples are found that suggest hyper-recombination phenomena involved in genome instability. The chloroplast genome (cpDNA) is circular, containing a pair of long inverted repeats [Fig. 1A, example of *Arabidopsis thaliana*; (Sato *et al.* 1999)]. Two structural isomers are present as an equimolar mixture (Palmer 1983), suggesting that frequent inversions occur during replication via hyper-recombination. Herpes simplex virus-1 (HSV-1) has a linear genome consisting of two unique sequences (U_L_ and U_S_) flanked by inverted sequences *ab*-*b'a’* and *a'c’*-*ca*, respectively (Fig. 1B). Four structural isomers are formed by inversions of the two unique sequences and are also detected at equimolar ratios (Bataille & Epstein 1995), suggesting that free inversion should occur between inverted repeats. However, the mechanism responsible for hyper-recombination phenomena resulting in frequent inversions remains unknown.

We previously observed very frequent inversion in designed amplification systems based on double rolling circle replication (DRCR) (Watanabe & Horiuchi 2005). Free inversions occurred in the inverted array of intrachromosomal amplification products. The DRCR process was first experimentally confirmed for amplification of the yeast 2-μm plasmid (Broach & Volkert 1991). This plasmid encodes a site-specific recombinase, Flp1p, and contains a pair of inverted repeats (599 bp) containing Flp1p recombinase target (*FRT*) sites. Flp1-*FRT* recombination produces equal amount of two structural isomers (Fig. 1C) and can initiate DRCR if Flp1-*FRT* recombination occurs just after either *FRT* site is replicated, as shown in Fig. 1D. Interestingly, Jayaram & Broach (1983) found very frequent inversion of Tn*5* inserted into 2-μm plasmids, and Jayaram (1986) later showed that whereas two-isomer formation is Flp1-*FRT* dependent, the Tn*5* inversion is *RAD52* dependent. The bacterial transposon Tn*5* (Jorgensen *et al.* 1979) consists of a unique central region flanked by a pair of inverted IS*50* components; inversion of this central region is an extremely rare event in *Escherichia coli* (Weber *et al.* 1988b). However, they found that inversion was extremely common, when Tn*5* was inserted into the HSV-1 genome (Weber *et al.* 1988a).

To elucidate the relationship between DRCR and frequent homologous recombination, here we exploited 2-μm circular plasmid and yeast linear genomes under DRCR conditions to test whether the DRCR process activates not only inversion of Tn*5* but also deletion/duplication of a direct repeat. We found that, regardless of DNA forms, DRCR strongly activates all three types of recombinational events.

## Results

### DRCR-dependent activation of inversion of Tn*5* inserted into 2-μm plasmids

**Figure 2:**
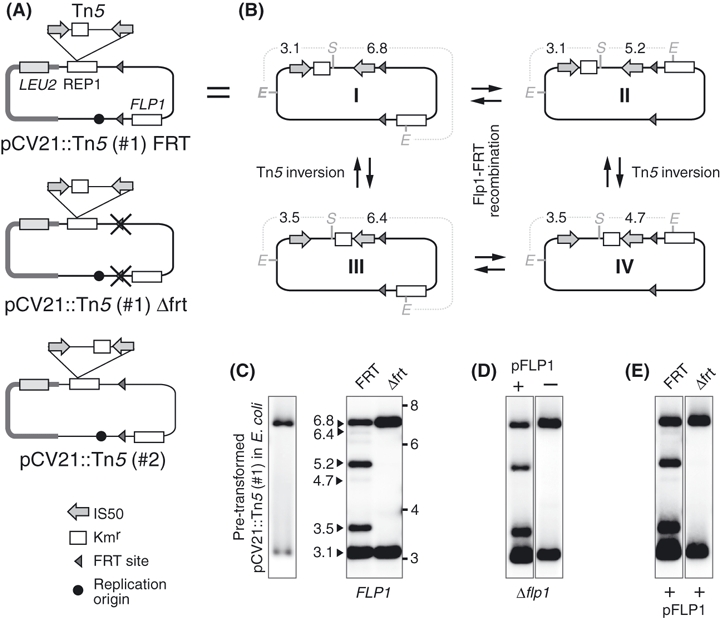
High frequency of inversion of Tn*5* transposed into the 2-μm plasmid. (A) Structures of two types of parental plasmids, pCV21::Tn*5* (#1) and pCV21::Tn*5*(#2). In both of them, Tn*5* is inserted into a similar site, but the orientation is the opposite. (B) Structure of four isomers, in which pBR322 and *LEU2* genes are omitted. They were derived from Flp1p-*FTR* recombination and Tn*5* inversion. The restriction sites (gray heavy lines; E: *Eco*RI, S: *Sal*I) and the sizes (kb) of fragments that hybridize with the IS*50* probe are shown. (C) Southern analysis of *Eco*RI*/Sal*I-digested plasmid DNA of a pCV21::Tn*5* (#1) in *E. coli.* (left, pretransformed) and a pCV21::Tn*5* (#1) (*FRT*) and its *frt* mutant (*Δfrt*) in yeast strain MRG5 (right) with the IS*50* probe. Agarose gel electrophoresis and Southern analysis were carried out as described in Experimental Procedures. (D) The same experiment as in C, except pCV21::Tn*5Δflp1* plasmid in yeast strains MRG5 in the absence or presence of pFLP1 plasmid. (E) The same experiment as in C, except the yeast strains MRG5 carried pFLP1 plasmid.

Plasmid pCV21 (Broach *et al.* 1982) is a 2-μm hybrid plasmid containing the bacterial pBR322 plasmid DNA and the *LEU2* gene (Fig. 2A). To construct a 2μm-based system with a pair of inverted repeats (IR), we transposed Tn*5* into pCV21. One of the resultant plasmids was designated pCV21::Tn*5*(#1) (Fig. 2A). A derivative from this plasmid, which had its *FRT* sites disrupted (see Experimental Procedures; Construction of *FRT* site-disrupted plasmids), was generated and designated pCV21::Tn*5*(#1)(*frt*^−^) (Fig. 2A). This plasmid should undergo normal replication but not DRCR. These plasmids were first multiplied in *E. coli* (*recA*^*−*^), extracted and then transformed into a yeast strain without the 2-μm plasmid (MRG5; cir^0^). The plasmid DNA was extracted, digested with *EcoR*I and *Sal*I, separated by agarose gel electrophoresis and analyzed by Southern hybridization using IS*50* as a probe. The results are shown in Fig. 2C–E. The digestion patterns of both pretransformed (*FRT*^*+*^ and *frt*^*−*^) plasmids appeared to be identical (3.1 and 6.8 kb; Fig. 2C and data not shown), indicating no detectable recombination in *E. coli* (*recA*^*−*^). In yeast, as shown in Fig. 2C, although the *frt*^−^ mutant plasmid showed the same digestion pattern of the pretransformed plasmid, the pCV21::Tn*5*(#1) produces multiple DNA fragments, six of which correspond to those derived from four structural isomers (form I to IV in Fig. 2B) produced by Flp1-*FRT* recombination and Tn*5* inversion. The low-density 6.4- and 4.7-kb bands are consistent with previous data by Jayaram & Broach (1983). The Tn*5* inversion was also blocked by *FLP1* disruption and restored by exogenous *FLP1* expression (Fig. 2D). However, blockade of Tn*5* inversion by *FRT* disruption was not reversed by *FLP1* expression (Fig. 2E). Another, independent, Tn*5*-inserted plasmid, pCV21::Tn*5*(#2) (the Tn*5* orientation opposite to that of the pCV21::Tn*5*(#1), Fig. 2A), also required intact *FRT* sites for Tn*5* inversion (unpublished data). It is known that DRCR depends on both the *FRT* site and the *FLP1* gene (Jayaram & Broach 1983). The present results show that the *cis* (*FRT* site) and *trans* (*FLP1* gene) elements are also required for Tn*5* inversion.

### Deletion and duplication are also activated by DRCR in 2-μm plasmids

**Figure 3:**
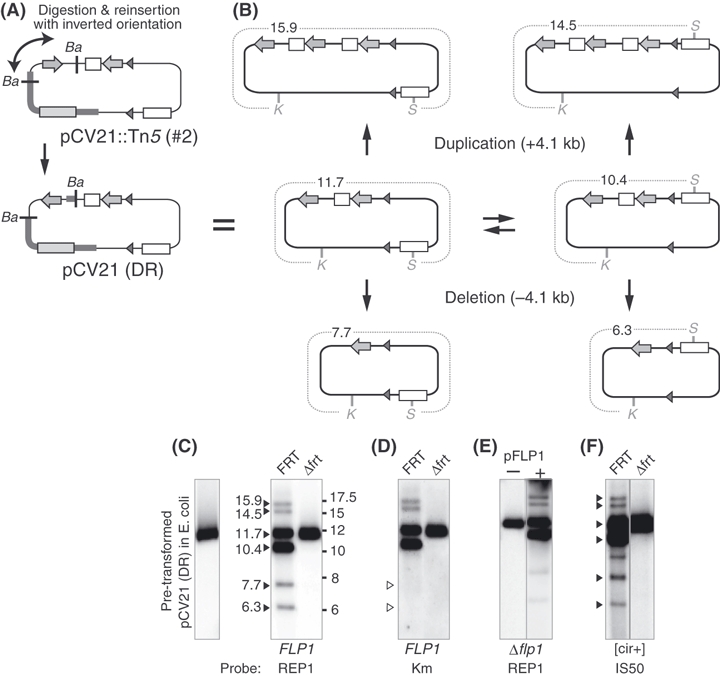
High frequency of deletion/duplication between direct repeats in the 2-μm plasmid. (A) Structures of plasmid pCV21::Tn*5* (#2) and pCV21(DR). For markers of the different structures, see Fig. 2. The methods of the construction of pCV21(DR) were carried out as described in Experimental Procedures. (B) Structures of six isomers are shown. They were derived from Flp1p-*FTR* recombination and deletion/duplication of direct repeats. The restriction sites (gray heavy lines; K: *Kpn*I, S: *Swa*I) and the sizes (kb) of fragments that hybridize with the *REP1* or IS*50* probe are shown. (C) Southern analysis of *Kpn*I*/Swa*I-digested DNA of pCV21(DR) in *E. coli.* (left, pretransformed) and a pCV21(DR) (*FRT*) and its *frt* mutant (*Δfrt*) in yeast strain MRG5 (right) with the *REP1* probe. Agarose gel electrophoresis and Southern analysis were carried out as described in Experimental Procedures. (D) The same experiment as in (C), except the Km^r^ gene was used as the probe. (E) The same experiment as in C, except pCV21(DR)*Δflp1* plasmid in yeast strains in the absence or presence of pFLP1. (F) The same experiment as in C, except MRG1 (cir^+^: 2-μm plasmid-containing yeast strain) was used as the host.

In addition to inversion, structural changes caused by homologous recombination also include deletion/duplication. To investigate whether DRCR activates deletion/duplication, we artificially inverted one of the two IS*50* of the pCV21::Tn*5*(#2) plasmid (Fig. 2A), constructing another 2-μm derivative plasmid with a pair of direct repeats (DR), designated pCV21 (DR) (Fig. 3A). We carried out similar experiments to those in the preceding section using *Kpn*I/*Swa*I digestion and *REP1*, Km^r^ or IS*50* probes. The plasmid DNA remains unchanged in *E. coli*, producing a single 11.7-kb band, but produced six bands in yeast, as shown in Fig. 3C. In addition to two main bands (11.7 and 10.4 kb) derived from two isomers produced by Flp1-*FRT* recombination, two lower (7.7 and 6.3 kb) and two higher bands (15.9 and 14.5 kb) were detected. This result can be explained, as shown in Fig. 3B, by 4.1 kb loss and gain by deletion and duplication via recombination between the direct repeats, respectively. In fact, the two lower bands did not hybridize with the Km^r^ gene (Fig. 3D), which is located between the direct repeats, indicating the presence of a deletion. The deletion/duplication was also detected using other restriction enzymes, including *Nde*I (data not shown). Furthermore, disruption of either the *FRT* site (Fig. 3C) or the *FLP1* gene (Fig. 3E) inhibited the deletion/duplication as well as DRCR. Finally, whereas *flp1* defectiveness was complemented by the *FLP1* gene (Fig. 3E), the *frt* mutant was not complemented by the *FLP1* gene (Fig. 3F). These results strongly suggest that inversion and deletion/duplication depend on DRCR processes of the 2-μm plasmid.

### DRCR induced in yeast linear chromosomes also activates deletion and duplication

**Figure 4:**
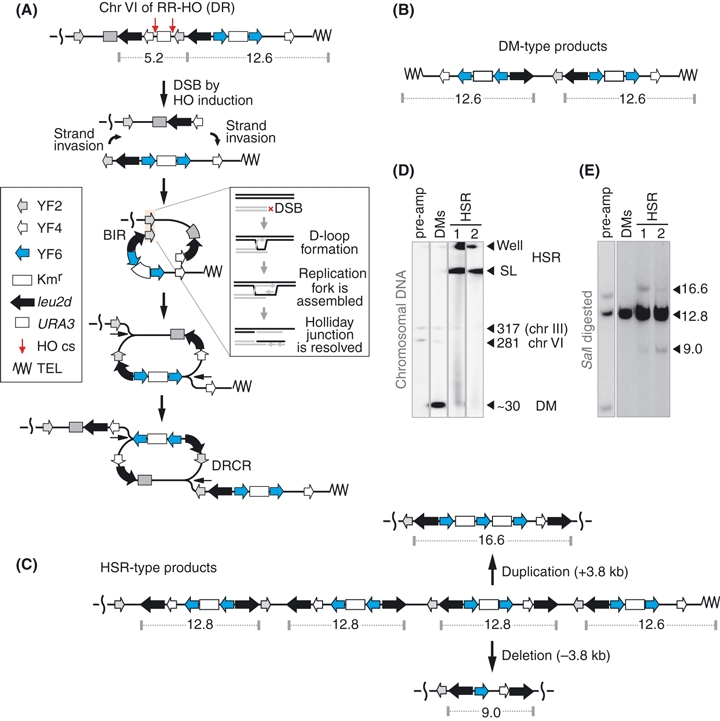
Double rolling circle replication (DRCR) induced on the yeast chromosome activates deletion/duplication between direct repeats. (A) DNA structure of pre-amplification clone and DRCR induction through DSBs (shown by red arrows) by HO endonuclease induction. This amplification cassette contains a pair of direct repeat of YF6 (blue arrow) and can be amplified via DRCR induced by BIR (break-induced replication) described previously (Watanabe & Horiuchi 2005). (B) DNA structure of DM-type product. (C) Three types of DNA structure, the expected, deletion and duplication, of homogeneous staining regions (HSR)-type product. (D) Pulsed-field gel electrophoresis (PFGE) and Southern analysis of a pre-amplification, one DM-type and two independent HSR-type samples. The *leu2d* gene was used as the probe. (E) Agarose gel electrophoresis and Southern analysis of *Sal*I-digested DNA of the samples from D with the *leu2d* probe.

Finally, to examine whether DRCR on the yeast linear chromosome can activate deletion/duplication, we constructed a DR structure (Fig. 4A and Fig. S1 in Supporting Information) within the amplification cassette described previously (Watanabe & Horiuchi 2005). This system contains two inverted pairs of genomic sequence, termed YF2 (gray arrow in Fig. 4A) and YF4 (white arrow). Following HO cutting, the cassette generates two chromosomal fragments, whose ends are designed to invade each other via the YF2 and YF4 sequences. This recombination-dependent replication process is known as break-induced replication (BIR). The double BIR is expected to induce DRCR (Fig. 4A). This system produces intra- and extrachromosomal products resembling products seen in higher eukaryotes, namely homogeneous staining regions (HSR) and double minutes (DMs), respectively. The resulting amplification cassette contains the Km^r^ gene (2.2 kb) flanked by a direct repeat of genomic nonspecific sequences termed YF6 (1.55 kb: blue arrow in Fig. 4A) and is located at the right terminus region of chromosome VI. The amplification marker *leu2d* (black arrow in Fig. 4A) has slight transcriptional activity and can complement leucine auxotrophy only if amplified. This strain lacks the native HO site and has a chromosomal HO endonuclease gene under the control of the GAL10 promoter (Butler *et al.* 1996). We plated approximately 1 × 10^5^ cells, a yeast strain with the amplification cassette unit containing DR structure (see Fig. 4A), on galactose plates lacking leucine to induce DRCR, as shown in Fig. 4A, and obtained 357 Leu^+^ colonies. Randomly selected 161 Leu^+^ clones were analyzed their genome structures using pulsed-field gel electrophoresis (PFGE). From these PFGE gel patterns, four HSR-type and 86 DMs-type clones were found. In addition, 60 colonies had chromosomal amplification products with lower copy number, and 11 colonies underwent Leu^+^ recombination between the *leu2d* gene and the original *leu2* fragment on chromosome III. The latter two types of Leu^+^ clones were described previously (Watanabe & Horiuchi 2005). Pulsed-field gel patterns of one DMs- and two independent HSR-type amplified clones (Fig. 4B,C) together with the pre-amplification control clone (the top structure in Fig. 4A) are shown in Fig. 4D. Next, *Sal*I-digested DNA from these samples was separated by agarose gel electrophoresis and hybridized with the *leu2d* gene as the probe (Fig. 4E). The control *Sal*I-digested sample produced 5.2- and 12.6-kb bands from the cassette on chromosome VI (Fig. 4A) and approximately 15-kb band from the *leu2* fragment previously used to disrupt an native HO site (Sandell & Zakian 1993). Neither DNA band appeared in the amplified samples, because the signal intensities of these single-copy DNA (at 5.2, 12.6 and approximately 15 kb) are much lower than those of the amplified *leu2d*. The *Sal*I digestion pattern of extrachromosomal products, which are formed not by DRCR, but by single BIR (Watanabe & Horiuchi 2005), showed a single 12.6-kb band. In contrast, the corresponding intrachromosomal products showed lower (9.0 kb) and higher (16.6 kb) DNA bands in addition to the 12.8-kb main band. This result can be explained by 3.8 kb loss and gain by deletion and duplication through the YF6 direct repeats, respectively, as shown in Fig. 4C. The lower band did not hybridize with the Km^r^ gene (data not shown), which is located between the direct repeats, indicating a deletion. These results strongly suggest that DRCR activates all three kinds of recombinational events, inversion, deletion and duplication, regardless of whether DRCR is induced on circular or linear DNA.

## Discussion

### DRCR is a recombinogenic process

Previously, we constructed a gene amplification system in yeast, in which DRCR induced by BIR produced two types of gene amplification product; both were analogous to two types of gene amplification products in tissue cultured cells, HSR and DMs. Moreover, we found that sequences flanked by inverted repeats in the HSR-type products randomly oriented. This means that recombination between the inverted repeats occurred freely (Watanabe & Horiuchi 2005).

Here, we investigated the relationship between the frequent recombination and DRCR. We constructed IR and DR structures in 2-μm plasmid DNA or yeast chromosome and examined whether inversion, deletion and duplication occurred under DRCR or normal replication. The results were clear-cut; all three types of recombination were extremely activated under DRCR, but not under normal replication. Thus, we conclude that DRCR itself is recombinogenic.

### What is the physiological function(s) of DRCR-dependent hyper-recombination?

When *leu2d* is used as an amplification selective marker, 10–20 copies of *leu2d* are enough to complement Leu^−^ auxotrophy (Watanabe & Horiuchi 2005). However, the actual copy numbers of *leu2d* in HSR-type products increase markedly to more than 100 copies, suggesting the existence of some mechanism stabilizing high *leu2d* copy number. One such possible mechanism is DRCR-dependent frequent inversion, because active inversions should destroy any very large palindromic structure that would be expected from deduced DRCR modeling, presumably resulting in stabilization of the amplified product. On the other hand, it has been generally observed that in cultured mammalian cells, the initial amplification unit (amplicon) is very long, but shortens, and the copy number increases drastically as selective pressure increases (Smith *et al.* 1992; Toledo *et al.* 1992; Ma *et al.* 1993). Indeed, in CHO cells, we observed that HSR-type products obtained through DRCR induced via the Cre-*lox* system were also extensively rearranged (Watanabe and Horiuchi, unpubl. data, 2010). However, the cause of such a drastic genomic rearrangement remains to be determined. It is well known that there is a large amount of repetitive sequences present in the higher eukaryotic genome, such as LINEs (17%) and retro-transposons (8%) in the total human genome (Krebs *et al.* 2009). Therefore, DRCR-dependent recombinogenic replication should contribute to the deletion of large unnecessary regions and leave only essential genes, resulting in shortened amplicons, increase in the copy number and stabilization of highly repetitive structures. Interestingly, in yeast, we have not found any intensive rearrangements in either HSR- or DMs-type amplification products. This can be explained at least in part by the fact that there are not so many repeated sequences, such as Ty elements (1.8%), in the total yeast chromosome as in the mammalian genome (Krebs *et al.* 2009).

### Recombinogenic DRCR model

**Figure 5:**
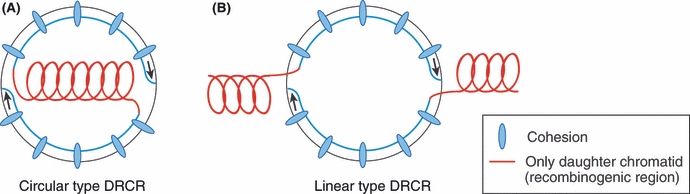
Double rolling circle replication (DRCR)-dependent recombinogenic model. (A) 2-μm plasmid-type DRCR. (B) linear chromosome-type DRCR. Arrow indicates replication fork, and blue oval structure indicates cohesin that bundles sister chromatids soon after replication. Red line indicates cohesin-free sister chromatid, which we designate the ‘only-daughter’ chromatid. See text in Discussion for more detail.

How does DRCR-dependent recombination become so activated? One possibility is that highly repeated structures produced by DRCR may be recombinogenic themselves. In fact, HSR products, for example, are highly repeated structures compared to unique sequence chromosomes, so that it should come as no surprise that the structure itself is recombinogenic. However, it is known that a single Tn*5* transposed into HSV-1 virus DNA inverts frequently (Weber *et al.* 1988a). It is hard to explain why a pair of inverted IS*50* (1.5 kb each) in Tn*5* transposed into the HSV-1 (150 kb) genome become so activated. Our model is shown in Fig. 5. In eukaryotes, it is well known that as replication proceeds, a protein complex, cohesin, bundles pairs of newly duplicated sister chromatids together until anaphase of the M period when the sister chromatids are separated from one another by proteolysis of the cohesin. Thus, one of the physiological functions of cohesin is to prevent two sister chromatids from separation (Nasmyth 1999). In addition, cohesin has a function in repair of DNA damage and transcription (Losada & Hirano 2005).

When DRCR initiates and proceeds in circular plasmids or chromosomes, two sister chromatids are produced and cohesin bundles them together, this being associated with replication in its initial stage. However, in the following stage, a pair of DRCR forks proceeds on the circular genome, as shown in Fig. 5A, because two replication forks chase each other. This results in one of the sister chromatids, the red strand in Fig. 5A, being forcibly separated from the other. Probably because cohesin unlinks physically, a resulting single chromatid, named only-daughter chromatid, which breaks loose from cohesin, emerges. The only-daughter chromatid produced by DRCR on a circular genome should be able to recombine freely, as shown in the above results, between repeated sequences on the only-daughter chromatid. DRCR induced on a linear chromosome, as shown in Fig. 5B, behaves similarly as in the case of the circular genome. In either case, until DRCR terminates, highly recombinogenic conditions would prevail. There are several lines of evidence supporting this model. One is a mutant of *rad21* (a component of cohesin in *Shizosaccharomyces pombe*) in which homologous recombination is stimulated approximately 10-fold (Grossenbacher-Grunder & Thuriaux 1981). Another is our own work (Kobayashi *et al.* 2004), in which we provided evidence that in yeast rDNA repeated clusters, both accumulation of extrachromosomal rDNA circles and loss of the *URA*^+^ marker inserted into rDNA significantly increased, approximately 9- and 4.2-fold, respectively, under cohesin-defective conditions. However, until now, because cohesin is essential for cell survival, it had not been possible to observe how cohesin-free sister chromatids behave in this context. If the model proposed here is correct, DRCR provides the first example of cohesin-free chromatids and cohesin should have an anti-recombinogenic function. We now investigate the relationship between DRCR and recombination proteins, such as Rad52.

### Other recombinogenic processes

Are there any other events in which DRCR is involved? Although not yet confirmed, DRCR may be involved in replication of cpDNA and HSV-1 DNA. The cpDNA has a DM-type structure and two isomers present in equimolar amounts, as shown in Fig. 1A (Palmer 1983). On the other hand, HSV-1 has a highly characteristic structure, as shown in Fig. 1B. The genome is linear, consisting of two unique fragments, U_L_ and U_S_, each of which is flanked by *ab* and *b'a’*, and *a'c’* and *ca* sequences, respectively. Interestingly, virus DNA in virus particles is mixture of four kinds of structural isomers (Bataille & Epstein 1995). These structures can induce DRCR, as shown in Fig. S2 in Supporting Information. DRCR can reasonably explain recombinogenic replication and finally produces four kinds of equimolar isoform. Interestingly, there are common properties in replication intermediate structures in cpDNA and HSV-1; in both cases, replication is associated with recombination. Replication intermediates are such complex structures that the majority immobilizes and stays in its original starting position during PFGE analysis, although replication intermediates are treated with a single-cut enzyme. The structure is reported to have a many-branched and entangled form (Bataille & Epstein 1995; Bendich 2004). These characteristics would be explained by DRCR-dependent recombinogenic properties (Fig. S2 in Supporting Information). Although a minority of cpDNA has a single copy of rDNA, replication intermediates are similarly complex to major cpDNA with inverted rDNA (Shaver *et al.* 2008), suggesting that the former may replicate in another mode, such as rolling circle replication (RCR) as discussed next.

Rolling circle replication (RCR) is another mode of replication analogous to DRCR. If our model is correct, RCR should also be recombinogenic, because it is similar to DRCR, which is expected to produce only-daughter chromatids. Yeast mtDNA may be a possible example of duplication through RCR, and there is a report that it is genetically recombinogenic (Dujon 1981). There are many other examples seeming to document replication via RCR, such as in Baculovirus (Martin & Weber 1997; Oppenheimer & Volkman 1997), telomeres of linear mtDNA producing t-circles (Tomaska *et al.* 2009) and recombinational hot spots (Hot) DNA in *E. coli* (Kodama *et al.* 2002). They raise interesting questions to be answered in the future.

In conclusion, we believe that there is little doubt that DRCR provides the genome with a previously unknown ability, namely a recombinogenic property.

## Experimental procedures

### Strains, plasmids, growth medium and cultivation

Yeast strains MRG1 (Ansari & Gartenberg 1997) and MRG5 (Tsalik & Gartenberg 1998) were provided by Dr. M. Gartenberg and used as the parental host strains. The genotype of MRG1 is *MAT*a, *ura3–52, leu2-*Δ1, *trp1-*Δ*63*, *his3-*Δ*200*, Δ*ade2*, *cir*^*+*^. The genotype of MGR5 is the same as MRG1 except for *cir*^*o*^ (Tsalik & Gartenberg 1998). Yeast strain LS20 was used for a host strain, into whose chromosome the amplification cassette was integrated (Butler *et al.* 1996). The following *E. coli* strains were used: MC1061 (*hsdR, mcrB, araD139, Δ(araABC-lue)7679, ΔlacX74, galU, galK, rpsL, thi*) (Casadaban & Cohen 1980), DH5α (F^−^, Φ80*dlacZ*Δ*M15*, Δ*(lacZYA-argF)U169*, *deoR*, *recA1*, *endA1*, *hsdR17(rK*^*−*^*, mK*^*+*^*), phoA, supE44,*λ^*−*^*, thi-1, gyrA96, relA1*) (Hanahan 1983) and XL10-Gold Ultracompetent Cells (STRATAGENE) (*Tet*^r^Δ(*mcrA*)*183*Δ(*mcrCB-hsdSMR-mrr*)*173 endA1 supE44 thi-1 recA1 gyrA96 relA1 lac* Hte [F′*proAB lacI*q*Z*Δ*M15* Tn*10* (Tet^r^) Amy Cam^r^]). The latter two strains were used as competent cells. Yeast 2-μm hybrid plasmid pCV21 was provided by Dr. J.R. Broach (Broach *et al.* 1982). To transpose Tn*5* into pCV21(Ap^r^, Tc^r^), we used bacterial plasmid pCHR381(Km^r^) (Sasakawa & Yoshikawa 1987), purchased from the CBS Fungal Biodiversity centre. It is temperature sensitive for replication and contains transposon Tn*5*. The pCV21 plasmid was transformed into *E. coli* strain MC1061 carrying a pCHR381 plasmid and Ap^r^ Km^r^ selected at 42 °C. Several hundred Ap^r^ Km^r^ colonies were collected, DNA was extracted, DNA samples were transformed into DH5α, Ap^r^ Km^r^ clones were selected at 42 °C, and two independent transposed clones were obtained. These were designated pCV21::Tn*5* (#1) and pCV21::Tn*5* (#2). Tn*5* was inserted into the long unique segment in both of these, as shown in Fig. 2A. Growth medium and culture conditions were described previously (Watanabe & Horiuchi 2005).

### Construction of *FRT* site-disrupted plasmids

pCV21::Tn*5* (#1) and (#2) DNA were partially digested with *Xba*I, and the single-cut product was separated from non- or double-digested product by agarose gel electrophoresis, extracted and purified using MonoFas DNA Purification Kit I (GL Science); the gap of the 5′-end was filled with KOD polymerase (TOYOBO), ligation was carried out with Ligation high Ver.2 (TOYOBO), DNA was transformed into *E. coli* using XL-10 Gold Ultracompetent Cells (STRATAGENE), and either *Xba*I site-disrupted plasmids were constructed. To disrupt another intact *FRT* site, the remaining *Xba*I site was completely digested, and similar procedures followed; two independent *FRT* site-disrupted plasmids, pVC21::Tn*5frt*^*−*^ (#1) and (#2), were generated.

### DRCR-dependent inversion assay for 2-μm plasmid with Tn*5* IR structure

Plasmid DNA to be assayed was transformed into yeast strain MRG5 [cir^o^] using Frozen-EZ Yeast Transformation Kit II™ (ZYMO RESEARCH), plated on SC, -Leu, 2% glucose plates and incubated at 30 °C. Colonies grown were cultured in 5 mL SC, -Leu, 2% glucose liquid medium for 40 h, and total DNA was extracted by the yeast DNA mini-prep method (Burke *et al.* 2000). Total DNA (approximately 25 μg) was digested with *Eco*RI and *Sal*I and was used in Southern hybridization.

### Construction of *FLP1* gene-disrupted plasmids

There is a unique *Swa*I site within the *FLP1* gene of the 2-μm plasmid. Thus, to disrupt the *FLP1* gene, pCV21::Tn*5* (#1) DNA was digested with *Swa*I and linker ligation was carried out. The following linkers were used: 5′-CTTACCCGGGTAACGATACAGTGGTACCTTACCCGGGACTT-3′; 5′-AAGTCCCGGGTAAGGTACCACTGTATCGTTACCCGGGTAAG-3′. The two linkers were annealed, digested with *Sma*I and ligated with *Swa*I-linearized 2-μm plasmid DNA (pCV21::Tn*5*), and an *FLP1*-disrupted plasmid was constructed, designated pCV21::Tn*5*Δ*flp1*. Using the same procedures, the pCV21(DR)Δ*flp1* plasmid was also constructed.

### Construction of pCV21(DR) in which a pair of IS*50* of Tn*5* is directly repeated

pCV21::Tn*5* (#2) has two *Bam*HI sites in pBR322 and the unique central region of Tn*5* (Fig. 3A). This plasmid was digested by *Bam*HI, and the two fragments were purified. The shorter fragment containing IS*50* was re-inserted into the larger fragment in the opposite orientation, generating pCV21(DR) (Fig. 3A). The same construction procedures were used to create the pCV21(DR) *ftr*^*−*^.

### Construction of *FLP1* gene expression vectors and expression of the *FLP1* gene

The *FLP1* gene encoded in pCV21 was amplified by PCR using 20-bp homologous sequences as primers. To clone the *FLP1* gene, the pSH47 plasmid (Guldener *et al.* 1996) was used. First, the Cre gene was removed by *Eco*RI and *Xho*I digestion of the pSH47 DNA, and then, the PCR products of the *FLP1* gene were cloned into pSH47 using the In-Fusion PCR Cloning System (Clontech). The resulting plasmid, termed pFLP1, was transformed into MRG5 containing the following plasmids: pCV21::Tn*5* (#1), pCV21::Tn*5 frt*^*−*^ (#1), pCV21::Tn*5*Δ*flp1* or pCV21::Tn*5* (DR)Δ*flp1* plated on SG-Ura-Leu plates. Colonies grown under selective conditions were purified, washed with distilled water, suspended in 3 mL of SC, -Ura, -Leu, 2% galactose liquid medium and cultured for 33–43 h to induce *FLP1* gene expression.

### Construction of a new amplification cassette with the DR structure

Plasmid pRR-HO, described previously (Watanabe & Horiuchi 2005), was used for making a new amplification cassette with a directly repeated (DR) structure, as shown in Fig. S1 in Supporting Information. To construct DR (YF6-Km^r^-YF6), the Km^r^-YF6 fragment was inserted into the 3′-side of the cassette plasmid (pRR-HO; Watanabe & Horiuchi 2005), as shown in Fig. S1A in Supporting Information. A pair of YF6 fragments (1.55 kb) has a DR structure, between which a Km^r^ fragment (2.2 kb) locates. The Km^r^ fragment, a derivative of transposon Tn*5*, consists of the Km^r^ gene and part of an IS*50,* as shown in Fig. S1A in Supporting Information.

First, the plasmid (pCV21::Tn*5*(#1)) was digested by *Xho*I and *Sal*I, and the Km^r^ fragment derived from Tn*5* was cloned into a *Sal*I site of the plasmid vector pBluescript SK+ (STRATAGENE) generating pBS-Km^r^ (Fig. S1A in Supporting Information). The single *Bgl*II site of pBS-Km^r^ was digested by *Bgl*II and disrupted with Blunting High (TOYOBO). On the other hand, the YF6 fragment was amplified by PCR (PrimeSTAR Max DNA Polymerase; TaKaRa) using pRR-HO as the template and In-Fusion primers as the primer (Fig. S1A in Supporting Information). The YF6 fragment was cloned into a *Sal*I site of pBS-Km^r^ (Bg^−^) using the In-Fusion system, generating pBS-Km^r^-YF6 (Fig. S1A in Supporting Information). This vector was used as a template, and PCR was carried out using Fw-primer containing an artificially produced *Bam*HI site sequence and Rv-primer containing a natural *Bgl*II sequence. The resulting PCR product was digested with *Bam*HI and *Bgl*II and purified using MonoFas DNA Purification kit I (GL Science) (Fig. S1A in Supporting Information). This fragment was ligated with *Bgl*II-digested and dephosphorized pRR-HO DNA, and pRR-HO DNA (DR) was obtained (Fig. S1A in Supporting Information). This plasmid was digested with *Bam*HI and *Bgl*II, and the linear DNA fragment with *Bam*HI and *Bgl*II ends (Fig. S1A in Supporting Information) was transformed into yeast using a Frozen-EZ Yeast Transformation kit II (ZYMO Research). Recipient strain was the LS20 strain, in which YF5-*lue2d*-YF4-*URA3*-YF2-*leu2d*-YF6-Km^r^-YF6 (YF2 (sequence position 257394-258454, of *Saccharomyces cerevisiae* chromosome VI, GenBank Accession ID, NC_-001138), YF4 (267165-268121), YF5 (262318-263257) and YF6 (263266-264862) are nonspecific sequences at the terminus region of chromosome VI) was integrated between the YF5 and YF6 fragments on the right terminus site of chromosome VI, generated and designated LS20RR-HO(DR). They were obtained under URA^+^ selective conditions, as shown in Fig. S1B in Supporting Information. The structure was confirmed by colony PCR [KOD FX (TOYOBO)] and Southern hybridization.

### PFGF and Southern analysis

All procedures were carried out essentially according to the methods described previously (Watanabe & Horiuchi 2005). DIG-labeled IS*50*, *REP1* or Km^r^ probes were prepared using the DIG labeling module (Roche).

### *GalHO* induction

Induction of the HO endonuclease gene was as described previously (Watanabe & Horiuchi 2005).

## Supporting Information / Supplementary material

The following Supporting Information can be found in the online version of the article:

**Figure S1** Construction of the chromosome VI with new amplification cassette.

**Figure S2** DRCR mode of HSV-1 genome.
