# Supplementary figures and images for: Double rolling circle replication (DRCR) is recombinogenic
Source: Genes Cells. 2011 May;16(5):503–13. doi: 10.1111/j.1365-2443.2011.01507.x (PMC3147054; doi:10.1111/j.1365-2443.2011.01507.x)

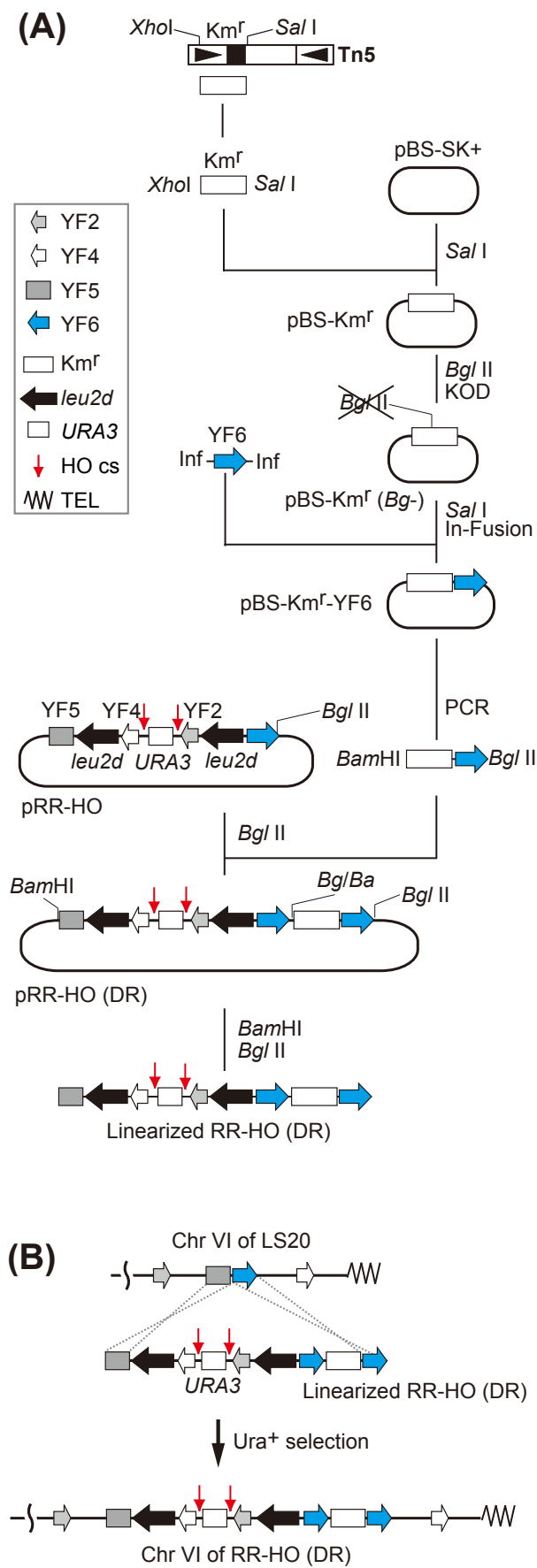

**Figure S1**

Supplement: Supplementary file 1 [file gtc0016-0503-SD1.pdf]

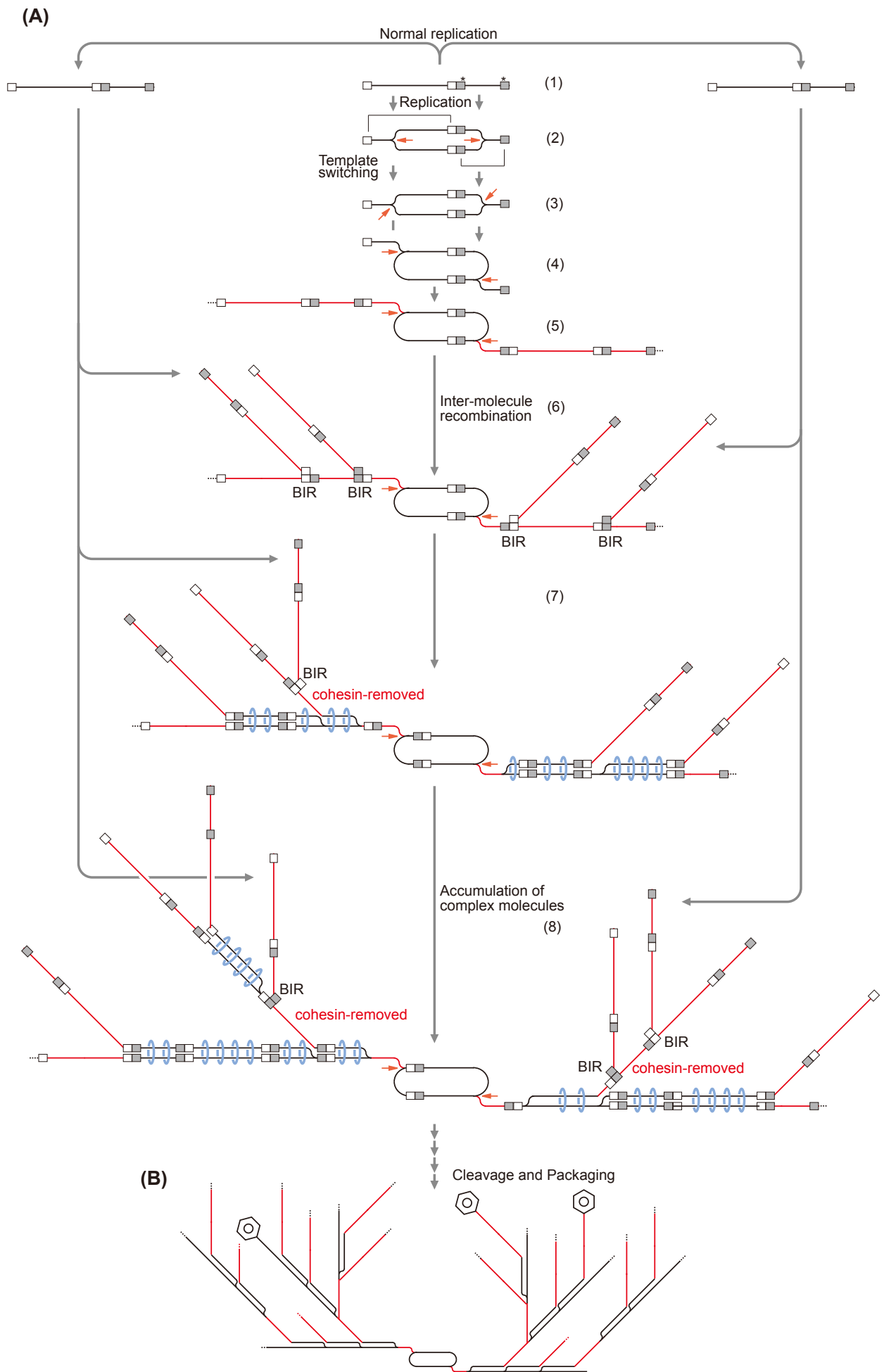

**Figure S2**

Supplement: Supplementary file 2 [file gtc0016-0503-SD2.pdf]
